# Impact of ketamine administration on chronic unpredictable stress‐induced rat model of depression during extremely low‐frequency electromagnetic field exposure: Behavioral, histological and molecular study

**DOI:** 10.1002/brb3.2986

**Published:** 2023-04-09

**Authors:** Moein Salari, Seyed Hassan Eftekhar‐Vaghefi, Majid Asadi‐Shekaari, Khadijeh Esmaeilpour, Somayeh Solhjou, Maryam Amiri, Meysam Ahmadi‐Zeidabadi

**Affiliations:** ^1^ Department of Anatomy, Afzalipour School of Medicine Kerman University of Medical Sciences Kerman Iran; ^2^ Neuroscience Research Center, Institute of Neuropharmacology Kerman University of Medical Sciences Kerman Iran

**Keywords:** depression, extremely low‐frequency electromagnetic field, ketamine, stress

## Abstract

**Objectives:**

In the study, we examined the effects of ketamine and extremely low‐frequency electromagnetic fields (ELF‐EMF) on depression‐like behavior, learning and memory, expression of GFAP, caspase‐3, p53, BDNF, and NMDA receptor in animals subjected to chronic unpredictable stress (CUS).

**Methods:**

After applying 21 days of chronic unpredictable stress, male rats received intraperitoneal (IP) of ketamine (5 mg/kg) and then were exposed to ELF‐EMF (10‐Hz, 10‐mT exposure conditions) for 3 days (3 h per day) and behavioral assessments were performed 24 h after the treatments. Instantly after the last behavioral test, the brain was extracted for Nissl staining, immunohistochemistry, and real‐time PCR analyses. Immunohistochemistry (IHC) was conducted to assess the effect of ketamine and ELF‐EMF on the expression of astrocyte marker (glial fibrillary acidic protein, GFAP) in the CA1 area of the hippocampus and medial prefrontal cortex (mPFC). Also, real‐time PCR analyses were used to investigate the impacts of the combination of ketamine and ELF‐EMF on the expression of caspase3, p53, BDNF, and NMDA receptors in the hippocampus in rats submitted to the CUS procedure. Results were considered statistically significant when *p* < .05.

**Results:**

Our results revealed that the combination of ketamine and ELF‐EMF increased depression‐like behavior, increased degenerated neurons and decreased the number of GFAP (+) cells in the CA1 area and mPFC, incremented the expression of caspase‐3, and reduced the expression of BDNF in the hippocampus but showed no effect on the expression of p53 and NMDA‐R.

**Conclusions:**

These results reveal that combining ketamine and ELF‐EMF has adverse effects on animals under chronic unpredictable stress (CUS).

## Graphical abstract

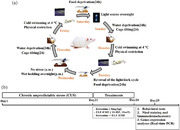

## 1 INTRODUCTION

Major depressive disorder (MDD), also recognized as depression, is a destructive psychic disorder (WHO, 2021) and is taken into consideration as one of the ten foremost reasons for incapacity worldwide (Verdonk et al., 2019). The control of MDD remains challenging because many do not respond to the standard treatment (Moretti et al., 2019). First‐line pharmacotherapies for depression, like the serotonin reuptake inhibitors (SSRIs), aim at monoaminergic systems but are not adequately effective according to limited by low response rate and slow therapeutic effects in the patients. So, they need weeks and even months of treatment (Akil et al., 2018; Gaynes et al., 2009). This postponement is especially concerning given the increased suicide rate in depressed people (Henriksson et al., 1993) and emphasizes a need for rapid‐acting antidepressants.

Research on ketamine to study its effects in the treatment of MDD took more than 10 years (Aan et al., 2012). A prominent discovery in the neurobiology of depression is the fast and steady antidepressant influence created by ketamine, an N‐methyl‐D‐aspartate receptors (NMDA‐R) antagonist is utilized to treat depression and suicide ideas (Ballard et al., 2014; Zarate et al., 2006). For the first time in 2000, it was stated that ketamine improves the indications of depression (Berman et al., 2000). The extensive use of electricity enhanced potential resources that led to alternating living systems (Boland et al., 2002). There are many reports of the effects of magnetic fields on biological systems. ELF‐EMF applies specific effects on brain performance, the activity of the nervous system, and cognitive functions (Goraghani et al., 2019; Sienkiewicz et al., 2005). A 10‐year follow‐up study on the relation between ELF‐EMF and human health that was performed by the World Health Organization (WHO) showed no unfavorable effect (WHO, 2007). However, it has been revealed that ELF‐EMF influences the central nervous system (CNS). The ELF‐EMF exposure (50 Hz; 1mT) can cause increasing in vivo hippocampal neurogenesis (Cuccurazzu et al., 2010). Dehghani‐Soltani et al. (2021) showed that EMF sensitizes glioma cells by modulating the expression of cyclin‐D1, p53, and O6‐methylguanine‐DNA methyltransferase. Also, several reports confirm that some frequencies' EMFs can induce p53 gene expression. P53, as a checkpoint gene, plays a critical role in various mechanisms, same as induction of apoptosis, cell cycle arrest, and differentiation (Ahmadi‐Zeidabadi et al., 2019; Amiri et al., 2018). Furthermore, EMF exposure can promote cell survival and suppress neuronal apoptosis in the nervous system (Oda et al., 2004). Also, chronic exposure (for 1 or 4 h) to ELF‐EMF (50 Hz; 1mT) Shows positive effects on spatial memory (Liu et al., 2008). Also, ELF‐EMF changes NMDA receptor action (Manikonda et al., 2007).

There are reports that dysfunction in the glutamatergic neurotransmission, particularly through NMDA receptors, plays an important role in the neurobiology and treatment of MDD (Garay et al., 2018; Kim et al., 2016). Brain‐derived neurotrophic factor (BDNF) is a momentous factor related to memory, neural plasticity, and emotional expression (Dwivedi, 2009; Kazemi et al., 2018; Minichiello, 2009).

We utilized the Chronic Unpredictable Stress Model (CUS) because it relates well to the period of depression in the clinic (Mutlu et al., 2012). The CUS model is the closest model to imitating stress in humans and is utilized to explain the interaction between deregulated biochemical signaling pathways and the psychopathological derangements related to stress (Chakravarty et al., 2013; Willner et al., 2002). The CUS model has a Worse impact on humans than the predictable stress model (Anisman et al., 2005; Umukoro et al., 2016; Willner et al., 2002). Furthermore, exposures to CUS have been reported to cause memory loss, loss of pleasure, anxiety, and depression (Anisman et al., 2005; Willner et al., 2002; Umukoro et al., 2016). Because of the usage of ketamine and ELF‐EMF for the treatment of MDD and the different effects on brain function, we inspected the impact of ketamine and ELF‐EMF on behavioral, morphological, and molecular changes in CUS rats.

## 2 MATERIALS AND METHODS

### 2.1 Animals

All experimental methods involving the use of animals were accredited by the Ethics Committee of the Kerman University of Medical Sciences. Our attempts were made to reduce animal's suffering and use the least number of animals (Ethics code: IR.KMU.AH.REC.1399.122). Forty adult male Wistar rats (200–250 g) were housed at 25 ± 2°C with a 12‐h light/dark cycle. The animals were kept in cages with four rats in any cage with free access to food and water. They were permitted 7 days to adapt to the animal room before the start of the experiments. All interventions were made between 9:00 and 17:00 h.

### 2.2 Chronic unpredictable stress (CUS) protocol

**FIGURE 1:**
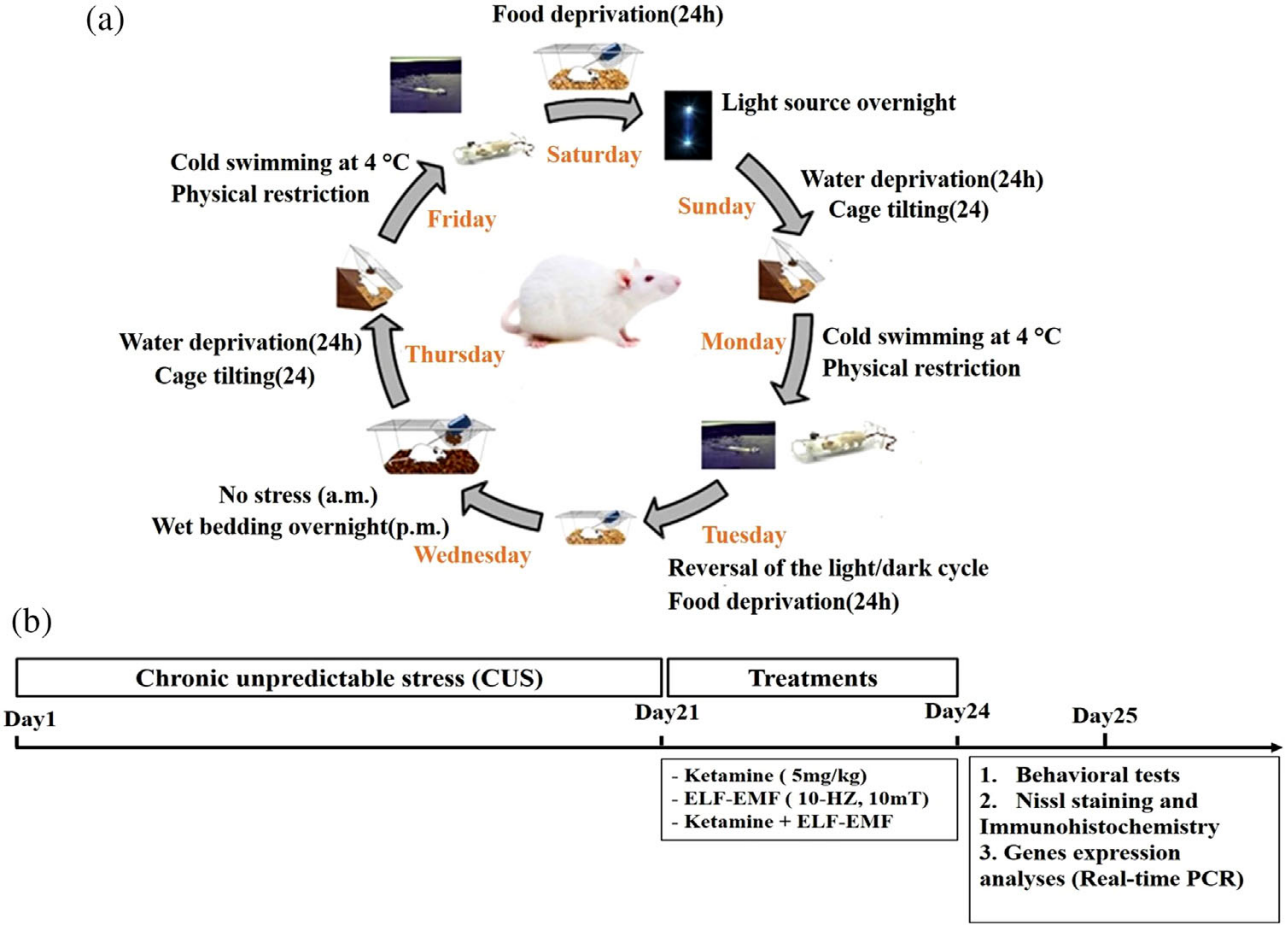
(a) The weekly protocol of the chronic unpredictable stress (CUS). Adult male Wistar rats are subjected every day to two stressors. (b) Experimental design of experiment. This weekly schedule is typically repeated over 3 weeks.

The animals in the CUS model group were separately housed and again and again exposed to a set of chronic unpredictable stressors as follows (1): cage tilting (24 h), overnight wet cage, swimming in water 4°C )5 min(, water and food deprivation (24 h), restricted movement (4 h) and reversal of the light/dark cycle. Two stressors were exerted per day, and the entire stress process lasted for three weeks in a random order (Figure 1a). The animals in the control group were housed in groups of four per cage without disturbing except for necessary procedures (cage cleaning or weighting) and had free access to food and water.

### 2.3 Experimental methodology

Animals were randomly allocated into five groups of eight (*n* = 8) animals. The normal group did not get any intervention. In stress groups, 24 h after the last stress exposure (day 21), animals obtained intraperitoneal (IP) administration of ketamine (5 mg/kg) once daily for 3 consecutive days (de Carvalho et al., 2019; Zhang et al., 2015) and then were exposed to ELF‐EMF (10‐Hz, 10‐mT exposure conditions) for 3 days (3 h per day) and 24 h after treatment, the behavioral assessments were performed (Figure 1b). Animals were weighed daily to compute doses of ketamine to be administered. Rats in all groups were sacrificed after the last behavioral test.

### 2.4 Electromagnetic field exposure device

A magnetotherapy instrument (Fisiofield Mini, Fisioline Co, Verduno, Italy) was utilized to produce EMF. This device can produce EMF varying from 0 to 100 Hz with an intensity of 0−10 mT. The square wave of EMF was created by a spherical electromagnetic prob. Two coils were located coaxially at a 160 mm distance from one another. In the EMF groups, animals were exposed to the electromagnetic field. Animals were located between the coils in a plastic cage. Based on previous studies, the magnetic field characteristics applied to the animals were selected. The animals were exposed constantly to the EMF of 1 groups 10 mT (10 Hz) for 3 days (3 h per day). The duty cycle of the square wave magnetic pulse was 50 %. Magnetic intensity was calculated utilizing the Gaussmeter GM08 device. The quantity of magnetic radiation would vary when the distance from the magnetic source alterations. So, based on our mathematical calculations, the efficient intensity would be virtually 10 ± 2 mT, which is in the range of moderate intensities.

### 2.5 Open‐field (OF) test

The open‐field test was applied to evaluate anxiety, general locomotor activity levels, and Tendency to explore animals (usually rodents) in scientific investigations. A box made of Plexiglas (80 × 80 × 50 cm) cm was used for OF test. The light, room temperature, and noise were controlled. Animals were positioned separately in the center of the box, and their motion was videoed for 5 min with an overhead camera. The data were examined utilizing a motion analysis program system (Noldus Ethovision® system, version 7.1, Netherlands). The total movement, number of grooming, and time spent in the center zone were measured.

### 2.6 Forced swimming test (FST)

The FST was done in 2 swimming trials using water containing a temperature between 22°C and 24°C, which animals cannot avoid. The first trial lasts 15 min. The second trial is performed 24 h later and lasts 5 min. FST behaviors were video‐recorded and examined by the researcher. Animal immobilization time in the second trial will be measured. Swimming is known as horizontal movements throughout the water surface. Immobility contains slight movements that the animal needs to keep its head above the water.

### 2.7 Morris Water Maze (MWM) test

MWM was utilized to analyze learning and memory. Rat utilizes spatial markers while swimming to find a submerged platform. The test box was a black round pool (160 cm diameter, 80 cm height 40 cm depth) and enclosed by visible signs obvious to animals. A round, submerged platform was situated below the water surface fixedly in the center of one of the four imaginary quadrants. Motion activities were recorded via the Noldus Ethovision system, version 7.1. For analyzing learning, three blocks were performed at a distance of 30 min from each other, and each block included 4 trials. During each trial, rats were accidentally released into one of the quadrants and allowed to swim for 60 s to a hidden platform position. The platform's location did not vary is the acquisition trials, and the animal was permitted to swim for 60 s to find the platform. In case of an animal did not find a platform in the 60s, it was picked up and put on the escape platform. Then, the distance moved and time spent to locate the hidden platform were examined. A trial was performed 2 h after the last trial to analysis the short‐term spatial memory. In this test, the platform was removed, and animals were permitted to swim freely for 60 s. The distance and time spent in the target zone were analyzed to measure spatial memory retention. All trials were done in 1 day between 10:00 AM and 5:00 PM.

### 2.8 Sample collection

After performing the last behavioral test, the animals of each group were sacrificed, and the brain was carefully removed from the skull. The right hemisphere was placed in 10% formaldehyde for histological staining. For obtaining fresh hippocampus tissue for molecular analysis, the left hemisphere is rapidly stored at –80°C.

### 2.9 Nissl staining

Nissl staining was utilized to analyze histopathological changes in neuronal morphology in the CA1 zone of the hippocampus and medial prefrontal cortex (mPFC). Summarily, paraffin was removed from the xylene and alcohols, stained in a 0.1% cresyl violet for 4 min, dehydrated in 95% and 100% alcohols, and cleared in xylene. Neurons were investigated in three microscopic fields, and cells with prominent nucleolus, observable round nucleus, and complete cytoplasm having visible and rich Nissl staining as viable neurons were counted. Neural cells with condensed cytoplasm and shrunken cell bodies were considered damaged neurons. The viable neuronal rate was calculated and reported as the percentage of the control group.

### 2.10 Immunohistochemistry

Paraffin was removed and rehydrated with xylene and descending series of ethanol. Brain slices were washed 2 **×** 5 min in twin 0.05%. For antigen retrieval, microwaves (340 W) and citrate‐buffer for 28 min. After incubating in 0.3% H_2_O_2_ solution for 30 min, and 0.1% Triton X‐100 for 10 min, the primary antibody (GFAP, Abcam, and 1:300 dilution) was added and incubated 12 h at 4**°**C. The secondary antibody was added at 4**°**C for 2 h, washed with 0.05% for 2 **×** 5 min, and DAB color solution was added. Finally, the sections were dehydrated with ethanol and mounted by Entellan. A total of three microscopic fields in each section were acquired in the mPFC and CA1 areas. The intensity of GFAP‐positive cells was quantified with the Image J program.

### 2.11 RNA isolation and real‐time PCR

RNA was extracted from hippocampal tissue by the guanidine isothiocyanate‐phenol‐chloroform method using RNX+ reagent (Cinagen, Tehran, Iran). The single‐strand cDNA was synthesized from total purified RNA using M‐MuLV reverse transcriptase and oligo (dT) primer (Cinagen, Tehran, Iran). Real‐time PCR reactions were done using Step One device (Applied Biosystems Foster City, CA, USA) by mixing 10 mL of innuMIX qPCR MasterMixSyGreen (Cinagen, Tehran, Iran). PCR amplification (40 cycles) was performed in the following way: initial denaturation for 3 min at 95°C, denaturation for 30 s at 95°C, annealing for 30 s at 55°C, extension for 40 s at 72°C, and final extension for 12 min at 72°C. The primer sequences were as follows: Caspase‐3, 5‐ AGCTGGACTGCGGTATTGAG‐3 (F), 5‐GGGTGCGGTAGAGTAAGCAT‐3(R); p53, 5‐CTACTAAGGTCGTGAGACGCTGCC‐3 (F), 5 TCAGCATACAGGTTTCCTTCCACC‐3(R); NMDA‐R, 5‐TTACCTTTGAGTCGCCCCTG‐3 (F), 5‐ CTGAGCAACGTCTGAGGGTC‐3 (R); BDNF, 5‐GACGACGACGTCCCTGGCTGA‐3 (F), 5‐ ACGACTGGGTAGTTCGGCACTGG‐3 (R). Glyceraldehyde‐3‐phosphate dehydrogenase (GAPDH) was amplified as an internal PCR control using the following primers: 5 ATGGAGAAGGCTGGGGCTCACCT‐3(F) and 5‐AGCCCTTCCACGATGCCAAAGTTGT‐3. The gene expression quantification was done using the REST 2009 program (QIAGEN, Hilden, Germany) and the 2^−ΔΔC^_T_ method.

### 2.12 Statistical analysis

Statistical analysis was done using GraphPad Prism 9 software. The Kolmogorov–Smirnov test was used to evaluate the normality assumption of behavioral, Immunohistochemistry, and histological data and real‐time PCR analyses. All values were presented as means ± standard error of the mean (SEM), and *p* values less than .05 was regarded as significant. In the MWM training in the acquisition phase, repeated measures two‐way ANOVA was utilized to analyze total distance, and escape latency to find the hidden platform. One‐way ANOVA was utilized to analyze all the data gathered from the forced swimming, MWM probe trials, open‐field, immunohistochemistry, molecular analysis, and histological staining. Dunnett test was used as post hoc analysis.

## 3 RESULTS

### 3.1 Immobility time in FST

**FIGURE 2:**
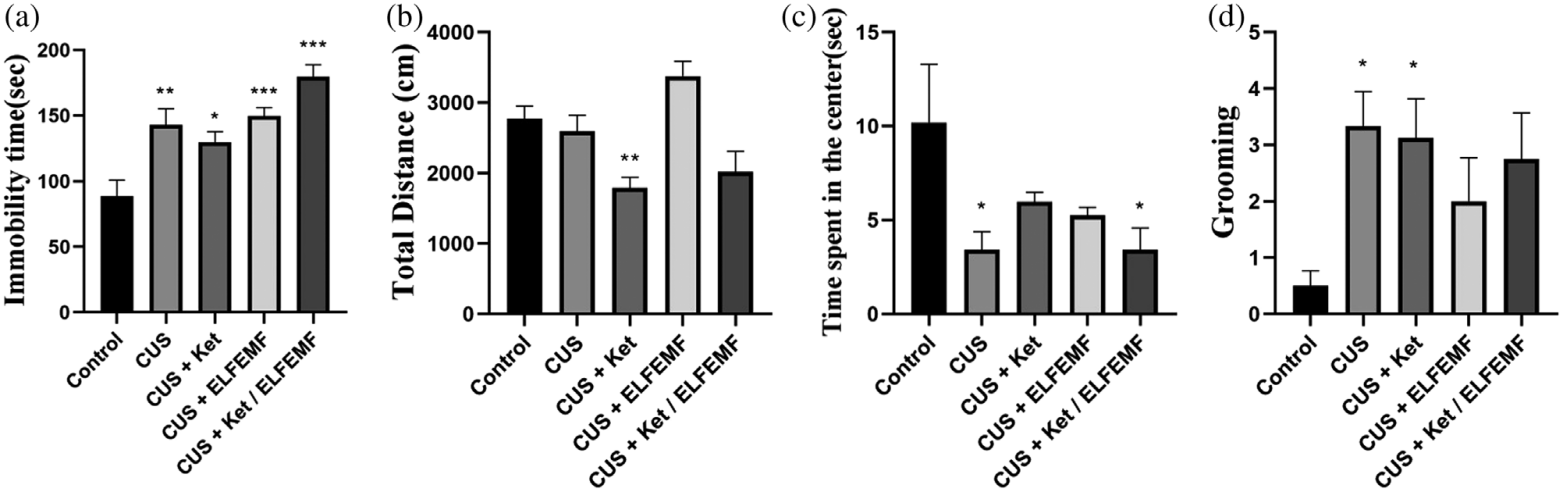
Effects of the combination of ketamine (5 mg/kg) and ELF‐EMF (10‐Hz, 10‐mT) in animals subjected to CUS. (a) The immobility time in the rat FST. (b) Locomotor activity in the open‐field test. (c) The time spent in the center in the open‐field test. (d) Number of grooming in the open‐field test. Each column represents the mean + SEM. Statistical analysis was performed by one‐way ANOVA, followed by Dunnett test. *p < .05, **p < .01, and ***p < .01 compared with the control group.

The FST is a standard behavioral hopelessness test to assess the rat's depressive‐like behaviors. Figure 2a shows the effect of ketamine and ELF‐EMF on immobility time in the FST. The results revealed that stress increased the immobility rate significantly compared to the normal group. Administration of ketamine alone and also exposure to ELF‐EMF increased immobility time. Also, combining ketamine and ELF‐EMF increased immobility time significantly more than in other groups.

### 3.2 Locomotor activity and anxiety behaviors in open‐field (OF) test

Animals were tested in OF for analysis of motor activity and anxiety‐like behaviors. Post hoc analysis showed that total travel distance was not significantly different between groups. Administration of ketamine alone decreased locomotion activity compared to the control group (Figure 2b). Time spent in the center (Figure 2c) and grooming (Figure 2d) was lower in the CUS group. Also, posthoc analysis indicates a combination of ketamine and ELF‐EMF decreased time spent in the center compared to the control group (Figure 2c).

### 3.3 Learning and memory in MWM test

**FIGURE 3:**
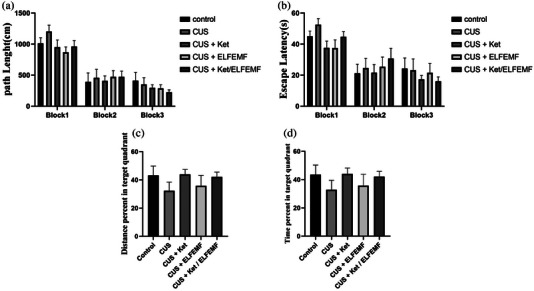
Effects of the combination of ketamine (5 mg/kg) and ELF‐EMF (10‐Hz, 10‐mT) in animals subjected to CUS in the MWM test. (a) Path length. (b) Escape latency in the learning phase (c) The percentage of time spent in the target quadrant. (d) The percentage of distance in the target quadrant in the memory phase. Each column represents the mean + SEM. Statistical analysis was performed by one‐way ANOVA, followed by Dunnett test.

MWM was utilized to evaluate spatial memory and learning. Learning in the MWM test was recorded as a decrease in the swimming escape latency and movement to the target zone. Comparison between CUS and control groups did not significantly differ in path length and escape latency. One‐way ANOVA exposed that treatment with ketamine and ELF‐EMF in path length and escape latency were no significant differences between the normal group in all three blocks of the learning phase (Figure 3a and b). A probe test (Figure 3c and d) was conducted 2 h after the last trial, and the value of time and distance in the target quadrant were examined to evaluate spatial memory retention. One‐way ANOVA revealed that there is no significant difference between all of the groups in distance and time in the target zone.

### 3.4 Effects of CUS and ketamine/ELF‐EMF treatment on morphology CA1 area of the hippocampus and mPFC neurons

**FIGURE 4:**
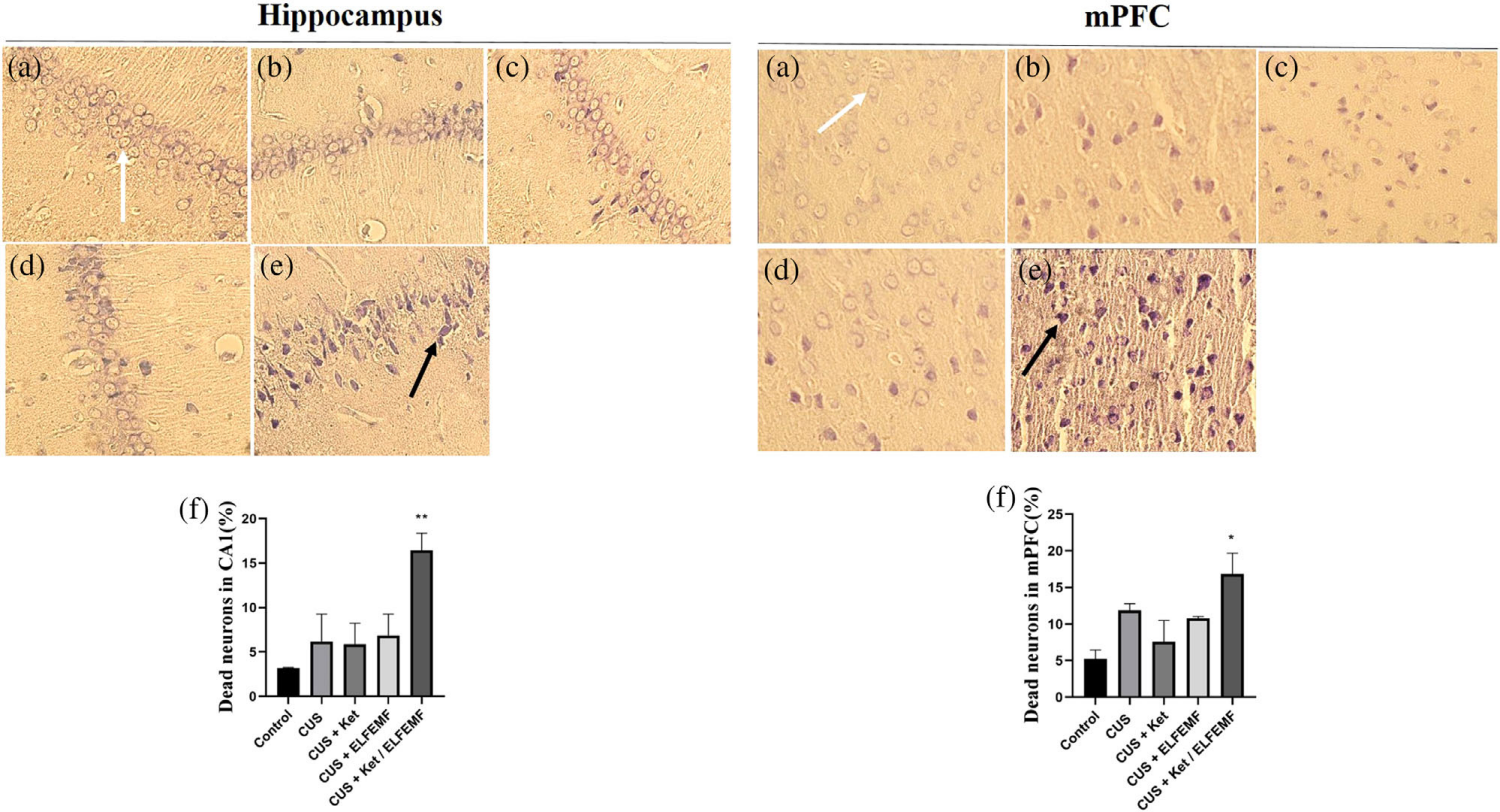
Intervention effects of ketamine and ELF‐EMF on CUS‐induced morphological changes in mPFC and CA1 area of the hippocampus (scale bar = 10 μm). Control group (a), CUS (b) group, CUS + Ket group (c), CUS + ELF‐EMF group (d), and CUS + Ket/ELF‐EMF group (e). White arrows show typical neurons and black arrows show damaged neurons .Each column represents the mean + SEM (f, g). Statistical analysis was performed by one‐way ANOVA, followed by Dunnett test. *p < .05.

Large and granular Nissl bodies in pyramidal neurons (mPFC and hippocampus) by Nissl staining. In the control group showed a larger number of mPFC and hippocampal neurons, regularly arranged, with normal morphology and clear cell boundaries (Figure 4a). In the CUS group, neurons with shrunken cell bodies and condensed cytoplasm were observed (Figure 4b) but did not significantly differ significantly from the control group. The combination of ketamine and ELF‐EMF (Figure 4e) increased the degeneration of neurons in the mPFC and CA1 area (Figure 4f) compared to the control group.

### 3.5 Effects of CUS and ketamine/ELF‐EMF treatment on expression of GFAP in CA1 area of the hippocampus and mPFC

**FIGURE 5:**
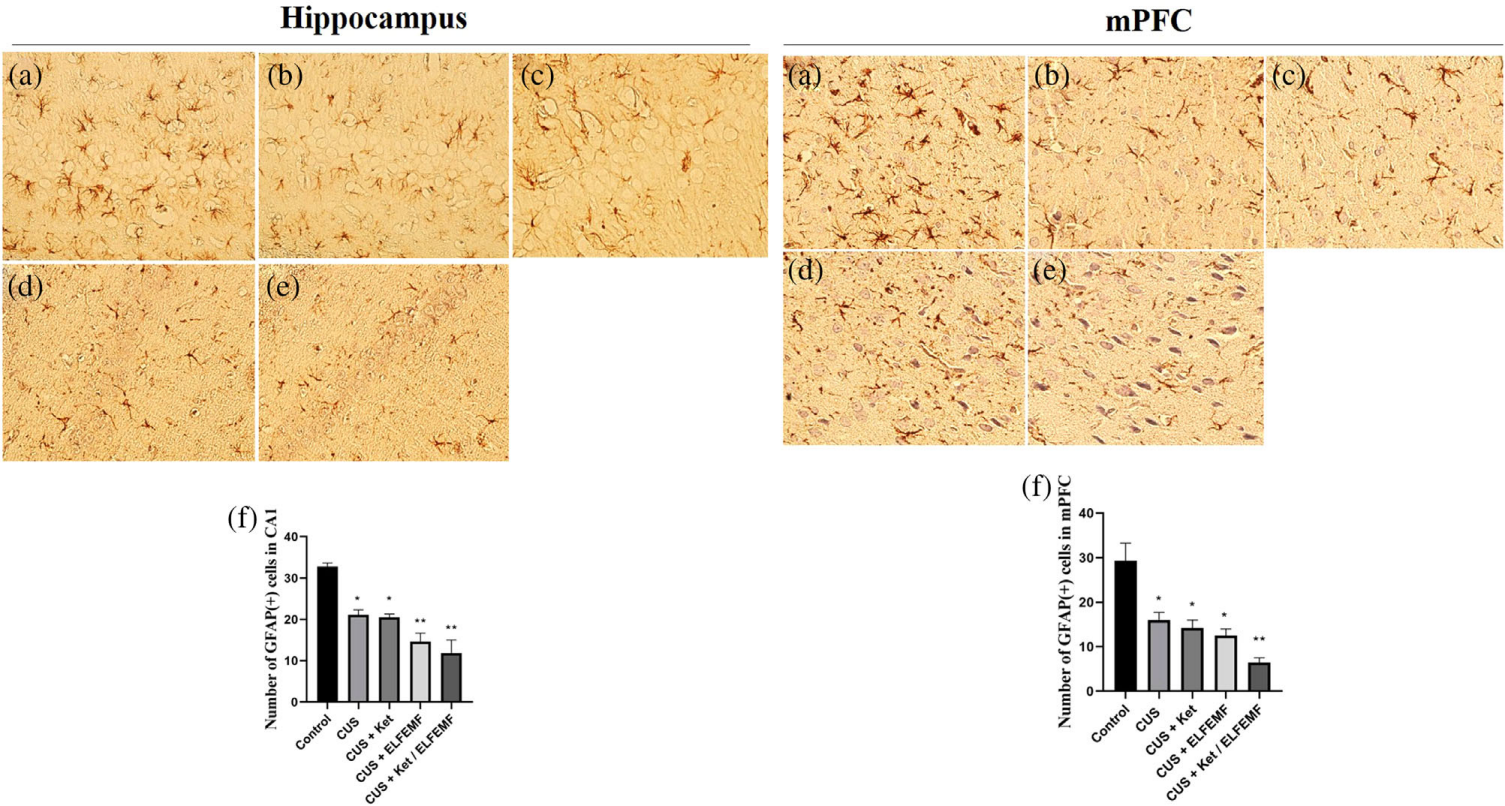
Change of GFAP expression in the CA1 area of the hippocampus and mPFC after CUS and ketamine/ELF‐EMF treatment in rat (scale bar = 10 μm). Images were shown for GFAP immunostaining in the control group (a), CUS group (b), CUS +Ket group (c), CUS + ELF‐EMF group (d), and CUS + Ket/ELF‐EMF group (e). Each column represents the mean + SEM (f, g). Statistical analysis was performed by one‐way ANOVA, followed by Dunnett test. *p < .05 and **p < .01 compared with the control group.

We showed that the CUS procedure caused significantly reduced GFAP expression in the CA1 area and mPFC (Figure 5b). Administration of ketamine alone (Figure 5c) and exposure to ELF‐EMF (Figure 5d) significantly decremented GFAP expression in the CA1 area and mPFC. Also, combining ketamine and ELF‐EMF (Figure 5e) decreased GFAP expression in both regions (Figure 5f).

### 3.6 Effects of CUS and ketamine/ELF‐EMF treatment on expression caspase‐3, p53, BDNF, and NMDA receptors in the hippocampus

**FIGURE 6:**
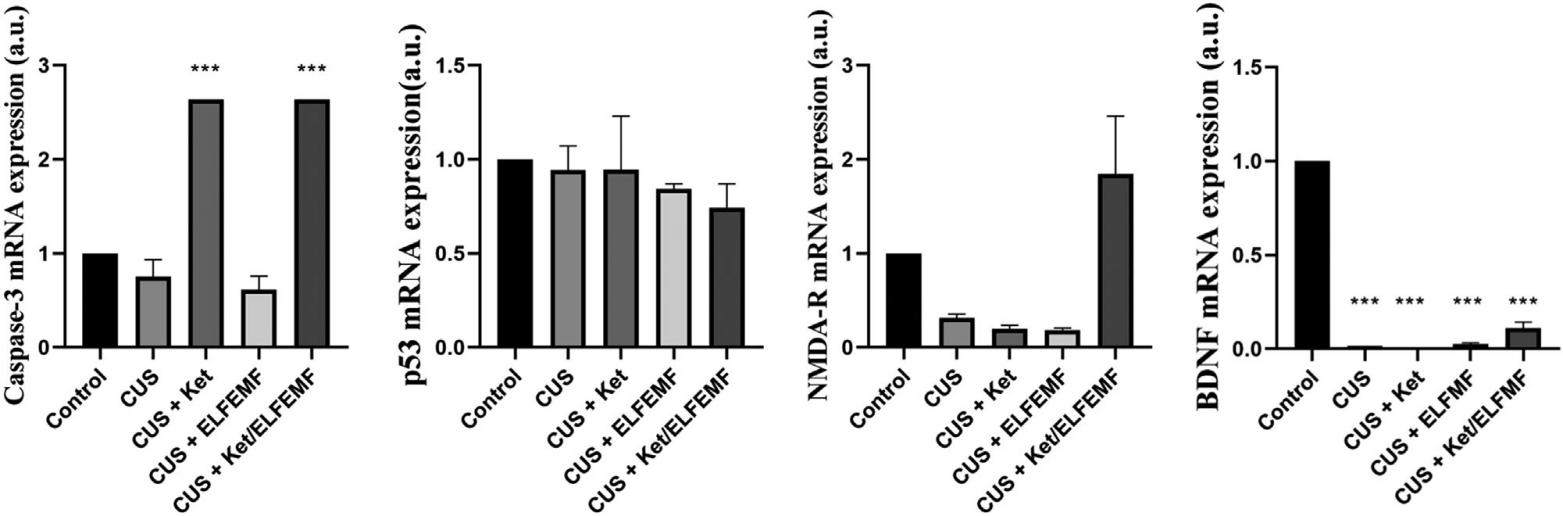
Effects of the combination of ketamine (5 mg/kg) and ELF‐EMF (10 Hz, 10 mT) in animals subjected to CUS on caspase‐3, p53, NMDA‐R, and BDNF expression. Each column represents the mean + SEM. Statistical analysis was performed by one‐way ANOVA, followed by Dunnett test. ***p < .001 compared with the control group.

Figure 6 shows the effect of the combination therapy with ketamine and ELF‐EMF hippocampal caspase‐3, p53, NMDA‐R, and BDNF levels. Analysis shows that ketamine alone increased caspase‐3 expression. Also, a combination of ketamine and ELF‐EMF causes increased caspase‐3 expression compared to the control group. The results revealed that stress significantly decreased BDNF expression compared to the normal group. Moreover, a combination of ketamine and ELF‐EMF significantly decreased BDNF expression (Figure 6). The one‐way ANOVA did not find significant differences in the p53 and NMDA‐R expression.

## 4 DISCUSSION

Major depressive disorder (MDD) is one of the most common cognitive disorders affecting approximately 300 million people worldwide (WHO, 2018), which leads to socioeconomic and individual impact (Greenberg et al., 2015). Depression is associated with suicide, one of the three main reasons for mortality for people ages 15−44 years (Aleman & Denys, 2014), with about 800,000 people committing suicide each year (WHO, 2018). The discovery of the fast and potent effects of ketamine has been praised as “arguably the most important discovery [in neuropsychiatric research] in half a century” (Duman & Aghajanian, 2012). Ketamine is the NMDA receptor antagonist that has the possibility to induce antidepressant effects in depressed patients in clinical trials (Berman et al., 2000). Due to the increasing use of electrical appliances, exposures to ELF‐EMF are significantly increased in both duration and intensity (Lacy‐hulbert et al., 1998). There are contradictions regarding the effects of ELF‐EMF on human health due to the duration and severity of the exposure. Recent research on the effectiveness of transcranial magnetic stimulation (TMS) and its type (r TMS) has shown that it is effective in treating anxiety and Treatment‐Resistant Depression (TRD) (Gross et al., 2007; Zwanzger et al., 2002) and FDA has approved it for the treatment of depressed patients . Though, it is hard to attain remission with rTMS alone.

Although MDD is a heterogeneous, multifactorial disorder, stress is an environmental risk factor related to disease onset (Krishnan & Nestler, 2008). The hypothalamus–pituitary–adrenal (HPA) axis is the principal stress‐responsive system. The activity of this axis is controlled by primary feedback from the hippocampus and negative feedback from forebrain structures (Belzung & de Villemeur et al., 2010; Herman et al., 1996; Jacobson & Sapolsky, 1991). It is valuable to create efficient animal models. We used the CUS model because CUS is the best model to imitate the low‐intensity, long‐term stress in human life. While in clinical settings, repeated doses of ketamine are used to treat MDD, most studies in rodents have used only single doses (Daly et al., 2018; Murrough et al., 2013; Wilkinson et al., 2018). For this reason, we used repeated doses of ketamine in this study. In the previous decades, psychopharmacological research mostly concentrated on molecules capable of moderating the glutamatergic system (Hashimoto, 2019; Lener et al., 2017). Ketamine, a glutamate NMDA receptor antagonist, plays an important role in synaptic plasticity and cognitive ability. Numerous studies have shown that ketamine exerts its fast antidepressant impacts by blocking NMDA receptors in inhibitory neurons. Followed by causes the disinhibition of pyramidal cells resulting in a burst of glutamatergic transmission (Duman, 2018; Krystal et al., 2019). ELF‐EMF affects the NMDA receptor by altering transient elevations in subunit expression (Li et al., 2014; Sienkiewicz et al., 2005) and ligand binding (Manikonda et al., 2007), but it does not affect their activities (Autry et al., 2011; Li et al., 2010; Zarate et al., 2006). In this study, we investigated the impact of the combination of ketamine and ELF‐EMF on behavior and brain histology.

Our data demonstrated that CUS augmented immobility time in the forced swimming test. Numerous reports show chronic stress increases immobility time in the TST and FST (Edem et al., 2022; Pałucha‐Poniewiera et al., 2021; Ren et al., 2022), which suggests depressive‐like behavior (Al‐Hasani et al., 2013; Zorkina et al., 2019).

In our study, there was a significant difference in immobility time between the control and stress groups which agrees with these previous findings showing behavior similar to depression in rats under CUS. Administration of ketamine alone and exposure to ELF‐EMF increased immobility time compared to the normal group. The results of a study showed that repeated injections of ketamine in the CUS model reduced immobility time in FST (Zhang et al., 2015). In another study, repeated administration of ketamine showed no change in immobility time in rats under chronic unpredictable stress (Jiang et al., 2017). It seems that the difference in study results is related to the dose of ketamine and the number of days of injection. Some reports have stated that ELF‐EMF is a mild stressor, which increases plasma corticosterone concentration (Ciejka et al., 2011; Manikonda et al., 2014; Mattsson et al., 2012). The result shows that a combination of ketamine and ELF‐EMF caused increased immobility time compared to the control group.

Results of our study demonstrated that stress augmented anxiety‐like behaviors in the open‐field test. There have been numerous reports of an association between anxiety and stress in animal models showing stress increases anxiety‐like behaviors in the open‐field test (Fitzgerald et al., 2021; Jiang Y et al., 2017). OF results indicated that the administration of ketamine reduced the total traveled distance. The combination of ketamine and magnetic field showed a significant decrease in time spent in the center zone compared to the control group.

Chronic stress has complex effects on cognition. The hippocampus is involved in spatial/relational memory (Kesner et al., 1987; Nunez, 2008). The Morris Water Maze (MWM) is the standard test in depressed animal models for examining memory and spatial learning (Aisa et al., 2007; Darcet et al., 2014). In humans and animals, chronic stress exposure is usually related to impaired cognitive task performance (Isgor et al., 2004; McEwen & Sapolsky, 1995; Sauro et al., 2003). Against this, other studies have reported that chronic stress exposure increases the acquisition or performance components of memory tasks (Shors, 2004). Our observations revealed stress did not affect cognitive ability in the MWM task. Also, the combination of ketamine and magnetic field did not impact learning and memory tasks.

The results of our study showed that CUS exposure causes a decrease in the density of astrocytes in the hippocampal CA1 and medial prefrontal cortex. Some reports suggest that inhibition of the number of astrocytes induces depressive phase behavior in rats (Banasr & Duman, 2008). As the main resident cells in CNS, which is a source of BDNF (Zhang et al., 2021), the antidepressant‐like effects of ketamine are related to microglia phenotypes (Yao et al., 2022). It was recently suggested that targeting microglia is for treating depressive disorders (Zhang et al., 2018) . In animal models, selective injury to astrocytes in the prefrontal cortex is adequate to induce depressive‐like behaviors (Banasr & Duman, 2008). GFAP is a cytoskeletal protein of astrocytes. It is commonly utilized as the main marker of astrocyte activation (Ren et al., 2022). A study of postmortem brain samples showed that GFAP‐positive cells in the CA1 region were significantly reduced in depressed patients. These reduced GFAP‐positive cells were related to astroglial changes in the hippocampus of depressed patients (Müller et al., 2001) that indicated abnormalities in astrocyte cells (Rajkowska & Miguel‐Hidalgo, 2007).In stress rats, the number and optical density of GFAP‐positive astrocytes were decreased in the dentate gyrus, hippocampus, and mPFC (Eldomiaty et al., 2020). In our study, an administration of ketamine alone and exposure to ELF‐EMF reduced the density of astrocytes in the CA1 area and mPFC compared to the control group. Also, the combination of ketamine and ELF‐EMF showed a decrease in the density of astrocytes in the CA1 area and mPFC. Furthermore, the combination of ketamine and ELF‐EMF increased the number of damaged neurons in both areas compared to the control group.

Based on the evidence, dysfunction of BDNF in the hippocampus is a primary event in the development and progress of cognitive depression pathology (Li et al., 2013). NMDA‐R is necessary for activity‐dependent BDNF expression (Chen et al., 2003; Tao et al., 1998) and BDNF downstream signal pathways. NMDA‐R modulators cause fast antidepressant impacts and increase BDNF expression, implicating NMDA‐R dysregulation in depression (Garcia et al., 2008; Li et al., 2011). Chronic stress decreases total hippocampal BDNF mRNA (Cieślik et al., 2011; Xiao et al., 2011; Zhang et al., 2014). Our data established that stress decreased the level of BDNF in the hippocampus. Also, ketamine and ELF‐EMF reduced BDNF levels. Studies in animal models show that chronic stress increases neuronal apoptosis via upregulation of caspase activity through decreased Bcl‐2 or increased caspase‐3 activity and downregulation of neurotrophic factors such as BDNF. Mild chronic stress in the hippocampus causes an increase in caspase‐3 and Bax‐positive cells (Liu et al., 2010). These effects lead to neuronal apoptosis and reduction of neurogenesis with induction of depressive‐like behaviors that eventually demonstrate the anti‐neurogenic and pro‐apoptotic effects of chronic stress (Kubera et al., 2011; Lee et al., 2020; Wigner et al., 2020). In our study, the administration of ketamine increased caspase‐3 expression, indicating a relationship with ketamine‐induced structural damage. Also, combining ketamine and ELF‐EMF increases caspase‐3 levels compared to the control group. Ketamine alters the apoptosis‐related gene in rat hippocampus (Li et al., 2018), which is consistent with the effects noticed in our study. Our data showed that ELF‐EMF did not affect caspase‐3 expression, which agrees with some studies' results. The basis for the contradictory results between our study and other studies possibly occur because of the difference in exposure setups, experimental conditions such as a static or alternative magnetic field, the intensity, frequency, and duration time of magnetic field, the time of recovery, study targets and assay methods.

## 5 CONCLUSION

Our study spreads information about the impact of ketamine and ELF‐EMF in a model of depression induced by CUS. We showed that combining ketamine and ELF‐EMF increased depression‐like behavior induced by CUS. An increase in degenerated pyramidal neurons accompanied this effect, a decrease in GFAP (+) astrocytes, decreased expression of BDNF, and increased expression of caspase‐3 but showed no effect on p53, NMDA‐R expression. This approach can open a new window for the treatment of depression disease, but more investigation is needed to complete therapeutic protocols in the future.

## CONFLICT OF INTEREST STATEMENT

The authors declare that they have no conflict of interest.

### PEER REVIEW

The peer review history for this article is available at https://publons.com/publon/10.1002/brb3.2986.

## Data Availability

The data sets are available from the corresponding author upon reasonable request.
